# Applicability Analysis of High-Voltage Transmission and Substation Equipment Based on Silicon Carbide Devices

**DOI:** 10.3390/mi16111192

**Published:** 2025-10-22

**Authors:** Huiyuan Zhang, Ming Nie, Qinxiao Dong, He Liu, Pengfei Jia, Zhiyuan Li, Yonghao Fang

**Affiliations:** 1 China Electric Power Research Institute, Beijing 100192, China; 2 State Grid Economic and Technological Research Institute Co., Ltd., Beijing 102209, China

**Keywords:** silicon carbide, power electronics, transmission and substation equipment, power semiconductors

## Abstract

Power electronics is an important feature of the new power system. The high-speed development of the new power system has gradually increased the requirements for high-efficiency and high-reliability high-voltage transmission and substation equipment. Silicon carbide (SiC) devices, which have the advantages of fast switching speed, low power loss, and high-temperature resistance, are the key core technology for the upgrading of high-voltage transmission and substation equipment. The paper begins by examining the evolution of the demand for high efficiency, compact and reliable power electronic devices for high-voltage transmission and substation systems at different stages of energy development. SiC has emerged as a key technological path to break through the physical limits of silicon-based devices, due to its outstanding material properties. Silicon-based devices encounter significant bottlenecks in high-voltage and high-frequency applications, with high switching losses and a junction temperature tolerance that is typically limited to 150 °C. In contrast, SiC devices can reduce switching losses by 60–80% and operate stably at temperatures up to 200 °C or even higher, thereby significantly enhancing system efficiency and power density. Finally, the paper provides a systematic analysis of the application of SiC devices in high-voltage transmission and substation equipment, exploring and identifying the technical bottlenecks and future research directions for SiC-based high-voltage transmission equipment.

## 1. Introduction

In the context of the deep change in global energy structure and the continuous rise in power demand, smart grid, as the core of the modern power system, has become an important guarantee for the high-quality economic and social development of its safe, stable and efficient operation. Power electronics technology, with its excellent power conversion, precision control and system optimization capabilities, has become a key technology driver to promote the innovative development of the smart grid [1]. It has also become the core support force of the modern energy conversion system, which is of great significance to support the clean and low-carbon transformation of energy and promote the construction of new power systems.

With the vigorous development of new energy generation, the smart grid, the new power system for high-voltage, high-power electronic modules and its performance demand shows a continuous growth trend. Power semiconductor devices, as the core components of power electronic devices, are related to the overall effectiveness of the system [2]. The silicon-based devices used in traditional power electronic devices have been gradually approaching the physical limits of the materials. In terms of voltage-level improvement, switching loss reduction and cost control, silicon-based devices are facing many technical bottlenecks. Silicon has a bandgap of approximately 1.1 eV and a critical electric field of about 0.3 MV/cm, while 4H-SiC has a bandgap of approximately 3.26 eV and a critical electric field of about 3.2 MV/cm [3]. Because of these intrinsic physical properties of the silicon limit, it is difficult to continue to improve the performance of power electronic devices only by improving silicon-based devices. It is not possible to meet the growing demand for new-generation power devices with high power density, high-temperature environmental adaptability and good switching performance [4,5,6].

In contrast, wide bandgap semiconductors (WBGs), such as silicon carbide, gallium nitride (GaN), diamond and gallium oxide (Ga_2_O_3_), exhibit inherent advantages due to their wide bandgap material properties. Among them, silicon carbide has developed rapidly in the field of power semiconductor devices, due to their excellent physical properties [7]. Compared with the traditional silicon-based semiconductors, silicon carbide devices show great application potential in the field of power electronics. It is especially suitable for the development of a new generation of high-end power electronic equipment and has an important strategic value for promoting the construction of the energy Internet [8,9]. It is essential to harness the technical merits of SiC devices and break through their remaining bottlenecks. This is crucial for original innovation in power electronics and for the next performance leap in transmission and substation equipment.

High-performance high-voltage power transmission equipment, as the core support of ultra-high voltage transmission technology, plays a key role in meeting the demand for flexible regulation of a high proportion of new energy access. Due to the new energy power generation randomness, volatility and grid voltage transient fluctuation problems are becoming more and more prominent. Higher requirements are put forward for the power transmission equipment’s transient voltage and current tolerance. At the same time, the new power system puts forward six major technology development directions for high-voltage transmission and substation equipment: high reliability, strong adaptability, depth of intelligence, green low carbon, autonomous and controllable. In this way, we can further promote the power system toward the direction of flexibilization, intelligence, transformation and upgrading of ultra-high voltage.

Based on the above background, this paper systematically combs through the evolutionary trend of the demand for high-efficiency, compact and reliable power electronic devices for high-voltage transmission and substation systems at different stages of energy development. The physical limits of silicon-based devices in high-voltage and high-frequency application scenarios are also analyzed. Meanwhile, the substitution advantages and application value of silicon carbide devices are explored. By systematically analyzing the adaptability of SiC devices in typical application scenarios, such as flexible DC transmission and power electronic transformers, and identifying the potential technical challenges, the current technical bottlenecks faced by high-voltage transmission and substation equipment based on SiC devices are further examined. In this way, we can look forward to the future direction of technological development, so as to provide practical guidance for the technological innovation of power electronics in the new electric power system.

## 2. Power Electronics

Power electronics technology is based on the on–off characteristics of semiconductor devices, relying on the coordinated operation of devices and electromagnetic circuits. Through precise control of switching timing and duty cycle parameters, the voltage level, frequency characteristics, waveform morphology and power factor are flexibly regulated to achieve the regulation, transformation, control and transmission of electric energy. In this way, we can achieve the dynamic regulation of voltage, current and power [10].

### 2.1. Power Electronics Development Process

In the actual power system, based on different application requirements, various types of power electronic devices are combined through the design of appropriate circuit and control strategies. Therefore, it is possible to form multiple circuit topologies, such as a rectifier, inverter, voltage regulator or frequency converter, which can further realize the flexible conversion of electric energy form [11].

Ideal power semiconductor devices need to have excellent static and dynamic characteristics. In the off state, they should be able to withstand high voltages. In the on state, they should achieve high current density transmission. Power semiconductor devices should also maintain a low on-state voltage drop. During the switching transition, it should have a fast on–off response and withstand high rate of change in the current (*di*/*dt*) and voltage (*du*/*dt*). Thus, switching losses can be kept low. In addition, good temperature stability is required.

**Figure 1:**
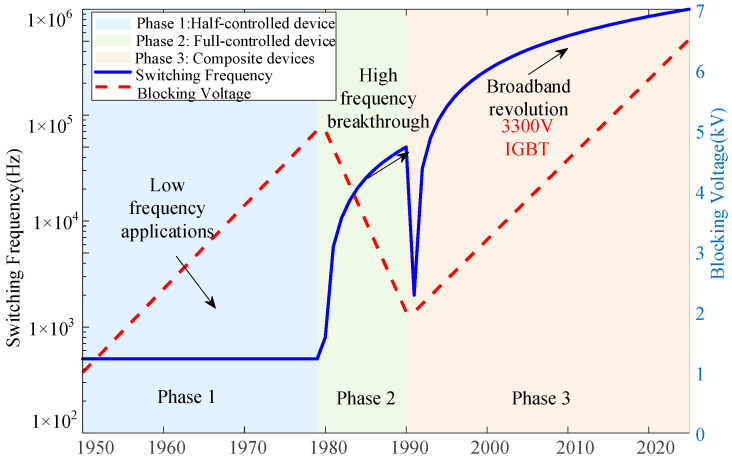
Development process of power electronics.

The development process of power electronic devices is shown in Figure 1:

As can be seen in Figure 1, power electronic devices, in accordance with the development, can be roughly divided into three stages [12].

The first stage is the era of semi-controlled devices (late 1950s–late 1970s). The representative device is the thyristor, which quickly replaced the mercury arc rectifier. It is mainly used for low-frequency high-power rectifiers, AC voltage regulators and circumferential wave converters.

The second stage is the era of fully controlled devices (late 1970s–early 1990s). The representative devices are power transistors, gate turn-off thyristors and power field effect transistors. These devices can be controlled by the gate signal to turn on and off, opening the door to high-frequency applications. They are widely used in switching power supply UPS, medium frequency induction heating, small and medium power inverters and motor drives.

The third stage is the era of composite devices and modern power semiconductors (late 1980s to the present). The representative device is the insulated gate bipolar transistor (IGBT). After the IGBT blocking voltage exceeded 3.3 kV, it was gradually widely used in voltage-driven, fully controlled devices, such as P-MOSFET and IGBT. Since then, it has further spawned the integrated gate commutated thyristor (IGCT). The typical switching frequency of the traditional gate turn-off thyristor (GTO) is 500 Hz, while the operating frequency of the IGCT and high-voltage high-current IGBT can be increased to 1~3 kHz, which significantly reduces the switching loss and system volume.

### 2.2. Classification of Power Electronic Devices

**Figure 2:**
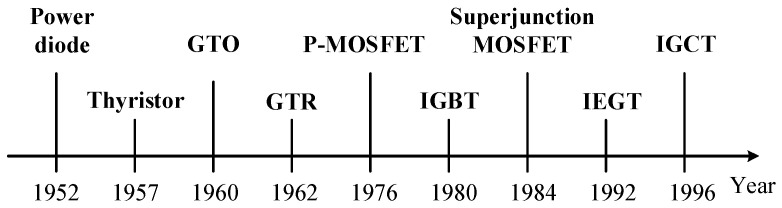
Development timeline of power semiconductor device.

In chronological order, the development timeline of the power diode, thyristor, gate turn-off thyristor (GTO), giant transistor (GTR), power field effect transistor (P-MOSFET), insulated gate bipolar transistor (IGBT), superjunction power field effect transistor (JFET), electron injection-enhanced gate transistor (IEGT) and integrated gate current converter thyristor (IGCT) are shown in Figure 2:

As can be seen from Figure 2, the current main power electronic devices can be summarized as follows [13,14]:

(1) Power Diode

Diodes work by conducting under forward voltage and cutting off under reverse voltage [15]. They are an uncontrollable device. Their conduction and shutdown can not be intervened by external control signals—only by the polarity of the voltage applied between the anode and cathode. These devices are suitable for rectifier circuits that do not require voltage regulation. They can provide continuity circuits for inductive loads, as well as having the ability to assume protective functions such as limiting, clamping and voltage regulation. Power diodes are also derived from fast recovery diodes (FRDs) and Schottky barrier diodes (SBDs).

(2) Thyristor

Thyristor conducting can be accurately triggered by the control signal, but the off state is dependent on external circuit conditions [16]. They are semi-controlled power devices. Since their introduction, their power handling capacity has been increased by a factor of nearly 3000. The highest single device power rating is (12 kV, 6 kA). Thyristors are also derived from the fast thyristor (FST), the light-controlled thyristor (LATT), the bi-directional thyristor (TRIAC) and others.

(3) GTO

The operating principle of GTO is to apply a positive gate pulse to turn on the device and apply a negative gate pulse to turn off the device [17]. GTO not only retains the advantages of thyristors, such as high voltage, high current, strong surge resistance and high cost performance, but also has the ability to self-shutdown. GTO is widely used in high-voltage, medium-to-high-power scenarios of chopper and inverter systems.

(4) GTR

GTR is a bipolar power transistor (BJT) which is capable of self-shutdown and is a current-controlled device [18]. It is easy to drive, and the collector-emitter turn-off is directly controlled by the base current signal. It is mainly used in the field of small and medium power converters, motor speed control systems and medium-frequency power supply devices.

(5) P-MOSFET

P-MOSFET belongs to fully voltage-controlled devices, and it has a high-frequency operation capability (up to MHz) level and a wide safe operating area (SOA). Its gate static resistance is of 10^9^ Ω level, and P-MOSFET has excellent temperature stability and no second breakdown phenomenon [19].

(6) IGBT

IGBT consists of P-MOSFET and GTR, which belong to voltage-controlled bipolar self-shutdown components [20]. It maintains the characteristics of fast-switching speed and high-operating frequency from MOSFET (Infineon Technologies AG, Munich, Germany). It also inherits GTR’s performance of low voltage drop in the on state, high voltage resistance, and high current resistance. Since its practical application in 1985, IGBT has become a mainstream power electronic device, dominating in the medium-voltage and medium-current range (10 kHz to 100 kHz) [21].

(7) JFET

JFET belongs to the majority of carrier devices, which were successfully developed in the 1970s. Its switching speed is extremely fast, and its static drive loss is low. However, the high on-state resistance of high-voltage power JFETs is high, which affects their application in the field of switching power supplies [22].

(8) IEGT

IEGT combines the advantages of IGBT and GTO. Its conductive characteristics are optimized by carrier injection enhancement technology. The electrode structure of IEGT is a flat plate crimping type, and the conduction saturation voltage is reduced. IEGT belongs to a fully controlled device, with a reduced conduction saturation pressure, wide safe operating region, high operating frequency, reliability and excellent heat dissipation performance [23].

(9) IGCT

The IGCT combines the advantages of MOSFET and a bi-directional thyristor (BCT). The core of the IGCT is an integrated gate driver with extremely low lead inductance. The IGCT is characterized by low losses, fast switching speeds and reliable shutdown. It can be used in converters from 300 kVA to 10 MVA without a series–parallel connection [24].

**Figure 3:**
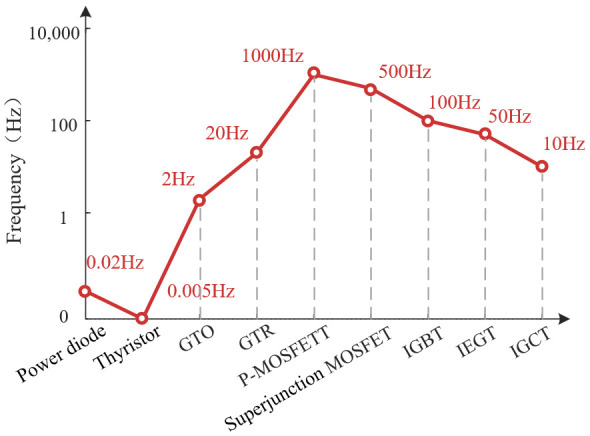
Typical switching frequency of power electronic devices.

**Figure 4:**
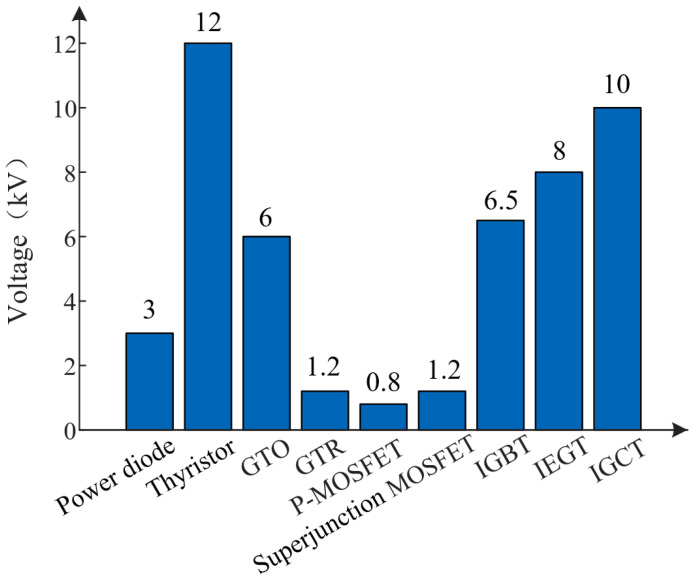
Blocking voltage levels of power electronic devices.

The typical switching frequencies and blocking voltage levels of the above power electronic devices are shown in Figure 3 and Figure 4.

As can be seen from Figure 3 and Figure 4, at a voltage level below 600 V, silicon-based MOSFET occupies the mainstream. In the medium- and high-voltage applications (0.6 kV to 6.5 kV) of fully controlled devices, the silicon-based IGBT occupies a dominant position. However, silicon material has inherent physical limitations, such as its critical electric field and bandgap. The performance of Si-based devices has thus stabilized around 10^9^~10^10^ W·Hz [25]. Their voltage is capped at about 6.5 kV, and their maximum operating temperature is under 175 °C [26,27,28]. The space for further improvement is very limited.

Due to the intrinsic characteristics of silicon materials, traditional Si-based devices have encountered significant bottlenecks in terms of increasing power density, withstanding high-temperature environments and optimizing switching performance. It is difficult to meet the urgent needs of new power systems for higher performance power devices.

## 3. Technical Characteristics and Advantages of SiC Devices

### 3.1. Material Characteristics of Silicon Carbide

For power devices of the unipolar type, the on-resistance represents a fundamental material limitation, which can be expressed by Equation (1) [29].

(1) $$R_{d}=4U_{j}^{2}/\left(\varepsilon_{0}\varepsilon_{r}\mu E_{F}\right)$$

where *R_d_* is the on-resistance of the device; *U_j_* is the breakdown voltage of the device; *E_F_* is the critical breakdown electric field of the material; μ is the carrier mobility; and *ε*_0_*ε_r_* is the dielectric constant. The on-resistance can be reduced by increasing the critical breakdown field of the material. That is, wide-band semiconductor materials can be used.

Currently, the third-generation semiconductor materials represented by SiC are recognized as the next-generation ideal high-voltage power devices, by virtue of their excellent material properties [30]. The forbidden bandwidth, critical breakdown electric field and electron saturation drift rate can be used to measure the performance of power semiconductor materials. It is also a key determinant of the performance of power electronic devices [31]. Table 1 shows the key electrical characterization parameters of various semiconductor materials.

As can be seen from Table 1, there are polymorphs of SiC materials. Among them, 4H-SiC has become an ideal choice for the preparation of high-performance power devices due to its excellent comprehensive performance. From the point of view of material classification, 4H-SiC belongs to the same wide-band semiconductor family as gallium nitride. Gallium oxide and diamond belong to the ultra-wideband semiconductor materials, by virtue of their wider forbidden band width.

Further comparative analysis shows that the new semiconductor materials, such as 4H-SiC, GaN, gallium oxide and diamond, are significantly better than the traditional silicon materials in the above parameter levels. Among them, SiC-based semiconductors show many material advantages by means of relatively mature preparation process and industrialization system. SiC already benefits from a commercially mature value chain: 200 mm substrates with defect density ≤ 0.5 cm^−2^ and 100 mm thick 4° off-axis epitaxy with <1% thickness uniformity [32]. Foundry PVT crystal-growth tools achieve 80% localization and a global wafer output of 2.3 M 150 mm equivalent pieces in 2023 [33,34,35].

(1) In terms of forbidden bandwidth, SiC has a wide bandgap characteristic and 4H-SiC material has a forbidden bandwidth of 3.26 eV, which is about three times that of silicon material. According to the physical properties of semiconductors, the increase in forbidden bandwidth means that the energy threshold required for the valence band electrons to jump to the conduction band is significantly increased. In turn, the intrinsic breakdown voltage rises approximately to V_B_ ∝ E_g_^2. For 4H-SiC (E_g_ = 3.26 eV), the theoretical 1D limit is 180–200 kV, whereas Si (E_g_ = 1.12 eV) saturates at 11–12 kV under the same drift-length and doping conditions [36].
(2) In terms of thermal conductivity, SiC has excellent thermal conductivity performance. The thermal conductivity is about three times that of silicon materials. It not only significantly improves the thermal efficiency of the device, but also enhances the through-current capability. It can operate stably in extreme high-temperature environments. The upper operating temperature limit exceeds 500 °C, breaking through the limitations of silicon-based power devices in terms of temperature (below 150 °C).
(3) In terms of carrier transport properties, the 4H-SiC material has a high electron saturation drift speed and a low dielectric constant. Its electron saturation drift speed reaches two times that of silicon materials. This feature enables 4H-SiC devices to achieve faster switching speeds, which demonstrates excellent high-frequency operation. At the same time, the low dielectric constant can give 4H-SiC devices stronger chemical stability and radiation resistance. This ensures reliable operation in harsh environments.
(4) In terms of critical breakdown field strength, SiC has a high critical breakdown electric field strength; 3C-SiC is about seven times higher than that of Si materials and can withstand high voltage stress. At the same time, the insulating breakdown field strength of 4H-SiC is up to 10 times that of Si material. This property allows a higher impurity doping concentration with a thinner drift layer structure in device fabrication. The critical electric field of 4H-SiC (E_c_ ≈ 2.5 MV/cm) is 7–10 times higher than that of silicon [37]. This allows its non-punch-through drift layer to be both thinner and more heavily doped for a given blocking voltage. Further, 4H-SiC-based high-voltage devices have very low on-resistance and a higher on-current density.

These excellent material properties can further break through the upper performance limit of power semiconductor devices. In practical applications, the use of 4H-SiC devices can significantly improve the energy conversion efficiency and power density of the system, which shows great technical and economic value.

### 3.2. Classification of Silicon Carbide Device

**Figure 5:**
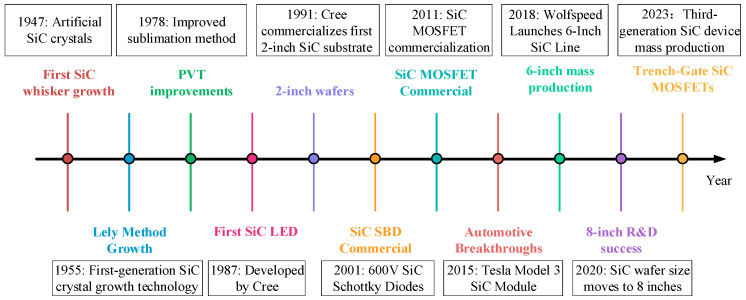
Development history of SiC-based devices.

Based on the excellent physical properties of SiC materials, silicon carbide power devices have become an ideal choice for replacing traditional silicon-based power devices. Its high voltage, high power, high frequency, high temperature and irradiation resistance characteristics can fully meet the application requirements of modern power electronic systems for extreme working conditions [38]. After extensive technical investigation, significant progress has been achieved in the development of SiC power devices. The development history of SiC devices is shown in Figure 5 [39,40].

**Figure 6:**
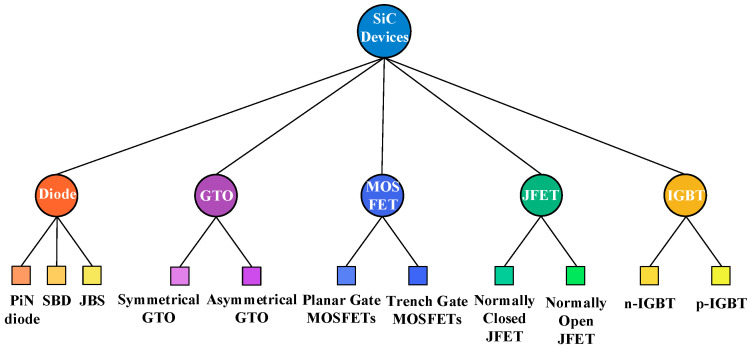
SiC device classification.

With continuous advancements in SiC substrate preparation and epitaxial growth technologies, such as PVT (physical vapor transport) boule growth and near-net-shape slicing, various types of silicon carbide power devices continue to emerge [41]. At present, a variety of silicon carbide products have been successfully developed. Silicon carbide devices are categorized, as shown in Figure 6:

As can be seen from Figure 6, SiC power semiconductor devices mainly include the following:

(1) SiC power diode

SiC power diodes mainly include the following: PiN diodes, Schottky barrier diodes (SBD) and junction barrier Schottky diodes (JBS). The JBS diode combines low loss characteristics of the forward conduction of the SBD diode and the low reverse leakage current characteristics of the PiN diode. It is widely used in blocking voltage scenarios up to 4.5 kV. The world’s first commercialized SiC Schottky diode was launched by 2001 Infineon. Currently, Cree, ST, Infineon and other manufacturers have introduced JBS products with a 0.6~1.7 kV voltage level and a maximum of 50 A current. Rohm has developed an SBD series with 0.6~1.2 kV and a maximum of 40 A current [42,43,44].

(2) SiC junction field effect transistor (JFET)

SiC JFET is a unipolar device. The principle of its operation is a unipolar conductive mechanism, which can be controlled by the gate PN junction depletion layer for switching. The voltage level is 0.6~10 kV. In 2008, SemiSouth released normally closed JFET products. During the same period, Rutgers University developed a normally closed SiC TI-JFET device with a breakdown voltage of 1.65 kV. In 2009, Sheridan’s team developed a 1.9 kV normally closed JFET. In 2012, Infineon launched the first commercialized JFET device [45]. The China Electronics Technology Group Corporation (CETGC) Institute (Nanjing, China) 55 developed 1.2~4.5 kV normally closed JFETs, based on their own SiC epitaxial materials [46]. Their maximum current can reach 25 A on a single chip. A 4.5 kV PiN diode, designed and tested by Zhejiang University, achieved a dc forward current of 2.8 A.

(3) SiC MOSFET

SiC MOSFETs are insulated gate unipolar devices. The absence of trailing currents during turn-off reduces switching losses and heatsink size. Their high-frequency switching characteristics can further reduce the size of the converter energy storage components and improve power density. In 2010, Cree and Rohm adopted a dual injection MOSFET (DMOSFET) technology route. In 2015, Cree proposed the center injection MOSFET (CIMOSFET) technology route. P-type injections in the JFET region below the gate further reduce resistance and the gate oxygen electric field. In the same year, Rohm developed trench gate MOSFET [47,48,49,50,51,52,53,54,55].

(4) SiC-insulated gate bipolar transistor (IGBT)

SiC IGBT replaces the N-type substrate with a P-type substrate. Its frontal active area process has compatibility with MOSFET. Therefore, SiC IGBT has high switching speed and low conduction loss characteristics. In 2014, AIST Japan developed injection-enhanced n-IGBT. The device can achieve a 20 A current output at a gate bias of 30 V, with a blocking voltage up to 16 kV. In 2015, Cree pre-treated SiC epitaxial wafers with a thermo-oxygenation process. A 27.5 kV IGBT device was developed [56,57]. In 2017, a bi-directional IGBT structure was reported by the Lunster Polytechnic Institute (Cork, Ireland). Two IGBT cells were realized in reverse parallel by integrating the gate oxygen and channel on the back of the device. The forward voltage drop is 9.7 V, and the voltage withstand capability reaches 7.2 kV [58,59].

(5) SiC gate turn-off thyristor (GTO)

GTO supports the dual injection of the anode and the cathode oligo mechanism. Higher-current densities can be realized, making it an alternative for high-current scenarios. In 2012, Cree developed SiC GTO with withstanding voltages up to 22.1 kV. In 2015, North Carolina State University developed the first symmetrical GTO with forward and reverse blocking, and withstanding voltages of up to 4 kV, which can support bidirectional power flow applications.

Currently, SiC devices are limited by factors such as manufacturing cost, mass production scale and reliability, and are mainly oriented to the low-voltage field for the industrialization layout. SiC devices are still 2.5 times more expensive than Si counterparts. While 200 mm wafer output reached 2.3 M equivalent pieces [60], with the upgrading and iteration of the R&D process, SiC SBD and SiC MOSFET have taken the lead in completing the industrialization of the technology. They began to enter the market as commercialized products. They not only achieve the gradual climb of voltage level covering 600 V−3.3 kV, but also significantly reduce the specific on-resistance (a falling from 15 mΩ·cm^2^ to 2.8 mΩ·cm^2^) [60]. This marks the SiC power devices formally stepping into the scale application stage.

**Figure 7:**
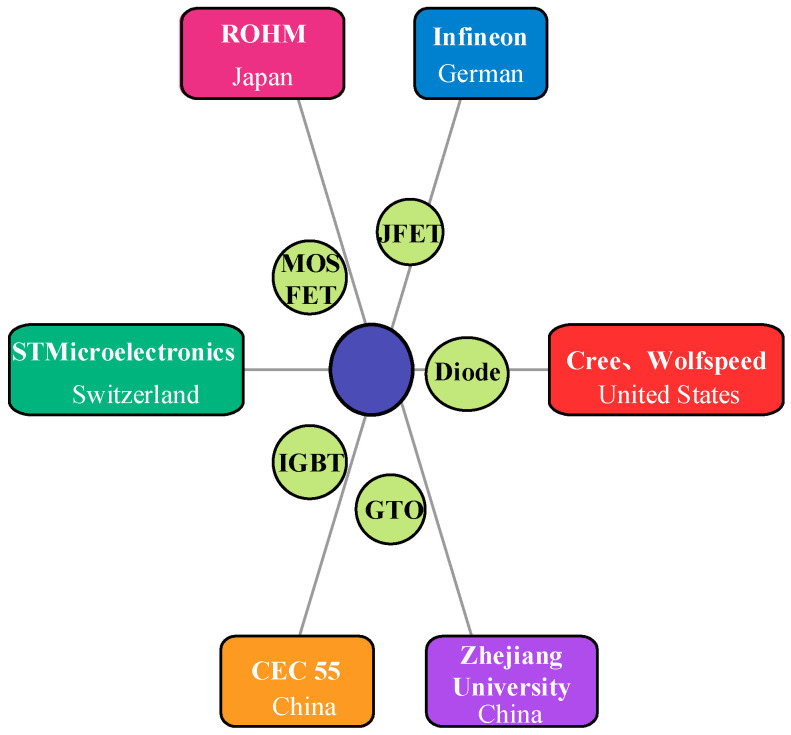
Major research and development centers of SiC device.

The main research and development centers of SiC devices are shown in Figure 7:

### 3.3. Applications of Silicon Carbide Device

**Figure 8:**
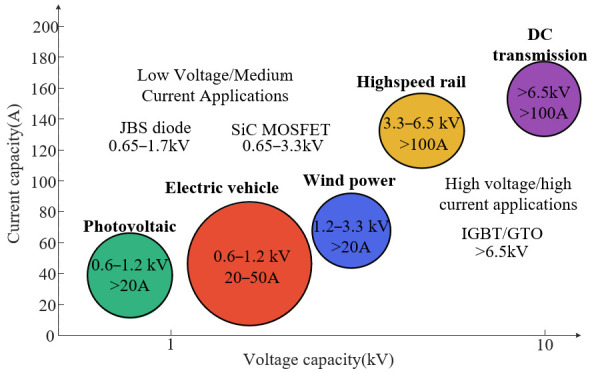
Electrical parameter requirements for SiC devices in different application scenarios.

From the point of view of the application scenarios of silicon carbide devices, they are mainly oriented to applications such as photovoltaics, electric vehicles, wind power generation, high-speed rail traction systems and direct current transmission. Different application areas have different demands upon the electrical parameters of the devices, as shown in Figure 8.

As can be seen from Figure 8, photovoltaic inverter modules are mainly applied to devices with 0.6~1.2 kV and current ratings greater than 20 A. Electric vehicle drive systems mainly apply devices with 0.6~1.2 kV and a 20~50 A current range [61,62,63]. Wind power converters mainly demand high-reliability devices with 1.2~3.3 kV and a current over 20 A. High-speed rail traction systems mainly apply devices with 3.3~6.5 kV and a current over 100 A. The high-voltage DC transmission field requires devices with a withstanding voltage that is more than 6.5 kV and an on-current that is more than 100 A, which requires IGBT or GTO support.

The technical characteristics and application areas of SiC devices are shown in Table 2.

As can be seen from Table 2, the SiC diode has the characteristic of no reverse recovery voltage compared to the Si-based diode. Among them, 0.65~1.7 kV JBS has the highest commercial maturity, with a single-tube chip current output of more than 100 A. This can be applied to the photovoltaic inverter, electric vehicle and wind power generation applications [64,65,66]. The PiN diode in the high-voltage, high-current field is limited by material defects and process complexity. Because thick-drift epilayers (>200 µm) still exhibit defect densities above 0.3 cm^−2^, the wafer yield remains < 60%. The marketization process is relatively behind [67]. It is necessary to reduce the defective density of the epitaxial layer of silicon carbide and develop the carrier lifetime enhancement process. The JFET device has a USCi cascade structure; the characteristics are similar to MOSFET. But the device gate drive has a weak regulation ability. In terms of SiC MOSFETs, 0.65–1.7 kV devices have gradually been promoted to mass-market applications. Applications have been realized in photovoltaic inverters, charging piles and wind power generation systems. With the continuous optimization of the gate oxygen process, MOSFET has been successfully applied to electric vehicles, and on other occasions, with stringent requirements for device stability. IGBT devices can further avoid the reliability risk of the gate oxygen structure (SiO_2_) under ultra-high-voltage and high-current operating conditions.

## 4. Analysis of Applications for High-Voltage Transmission and Substation

SiC devices have become an important direction for power electronics innovation by simplifying topology and improving energy conversion efficiency. Compared to the Si IGBT, the 1.2 kV SiC MOSFET enables a two-level inverter to achieve a peak efficiency of 99.2% at 20 kHz, versus 97.8% for its Si counterpart. It also halves the heat-sink volume and eliminates three snubber capacitors. On the other hand, while 6.5 kV Si IGBTs drop to 96.5% at 8 kHz, 6.5 kV SiC MOSFETs maintain 98.7% even at 15 kHz, allowing the ANPC topology to be simplified to a single bridge leg [68]. Currently, SiC devices have been scaled up in low-voltage electric vehicles and other fields. This section focuses on its applicability in high-performance, high-voltage power transmission equipment. The technical potential of SiC devices is explored in new high-voltage transmission scenarios for power systems.

### 4.1. Flexible DC Transmission System

During the operation of converter stations in flexible DC transmission systems, the power electronic devices in the valve manifolds are subjected to extreme operating conditions of volts and current. The current technical solution is to use a large number of silicon-based power modules in a series topology to achieve high-voltage conversion. This program can meet the high voltage requirements to a certain extent. However, the series connection of devices will lead to the difficulty of device voltage equalization control, high conduction loss, and a significant increase in system complexity and cost.

At present, the high-power electronic devices that are widely used in flexible DC transmission systems are dominated by silicon-based devices. The commutator valve that it constitutes generates a large amount of power loss during operation, which restricts the system’s energy efficiency improvement. The performance of such devices has approached the theoretical limit. The 650 V Gen-6 trench MOSFET achieves Ron = 2.8 mΩ·cm^2^ [69]. The technology iteration space is significantly narrowed. It is unable to meet the demand for higher-performance flexible DC transmission systems for new power systems.

Therefore, silicon carbide devices can be used to replace silicon-based devices in flexible DC transmission systems. The study of flexible DC power conversion units based on silicon carbide devices will cover key aspects such as the modular multilevel converter operating principle, half-bridge topology architecture, silicon carbide power device selection, capacitor configuration and drive circuit and protection circuit design.

In power electronic devices with voltage levels above ±500 kV, SiC power devices have significant differences from traditional silicon-based devices in terms of converter valve loss characteristics. For MMC valves, 10 kV SiC-MOSFET chips reduce the conduction loss to 0.8 W/A: a substantial improvement over the 3.2 W/A of 4.5 kV Si-IGBT. They also lower the total switching energy from 11 mJ/A to 4.8 mJ/A, under the conditions of 1 kHz and 125 °C. This improvement cuts the valve losses from 1.6% to just 0.8% of the rated power [70]. Consequently, the valve losses drop from 1.6% to 0.8% of rated power. The high-frequency switching characteristics of SiC power devices can effectively improve the energy conversion efficiency of modular multilevel converters and significantly reduce the overall power loss.

With the further development of new SiC IGBT, SiC GTO and other wide-bandwidth semiconductor devices, the voltage level and transmission capacity of the flexible DC transmission system will realize a significant improvement. It can provide a revolutionary technology path for large-capacity flexible DC transmission system, to a certain extent.

### 4.2. High Voltage DC Circuit Breaker

As a new type of equipment for integrating traditional mechanical switch and power electronic devices, the core function of high-voltage DC circuit breaker is to achieve fast and reliable breaking of the fault current. In this way, to ensure the safe and stable operation of the system, the high-voltage DC circuit breaker not only integrates the low conduction loss characteristics of mechanical switches and the high-speed control capability of power electronic devices, but also effectively overcomes the technical bottlenecks of traditional circuit breakers in terms of breaking speed, arc suppression and other aspects. Its main working principle is that the mechanical switch undertakes the task of current passage during normal operation, and the power electronic device quickly conducts during faults. With the current converter circuit to achieve current transfer and disconnection, it takes into account the low loss and fast disconnection capability [71,72].

At present, high-voltage DC circuit breakers are dominated by mechanical switching hybrid silicon-based IGBT devices. They have the advantages of fast breaking speed, arc-free operation, and a high operating frequency. However, there are still problems, such as high pass-state loss and the prominent over-voltage and over-current risk of single-tube devices. At the same time, the extensive access to distributed power sources in the DC transmission system and the diversified grounding methods lead to the diverse and complex system fault current’s characteristics. This further exacerbates the design difficulty of DC circuit breakers. The stringent requirements of the breaking capacity and response speed also pose a higher challenge to the system protection configuration.

Therefore, considering the characteristics of high withstand voltage, high power density, high switching speed, low switching loss and the fast response of silicon carbide devices, it can further be an ideal choice for large-capacity, high-voltage, high-frequency and high-temperature DC scenarios. By introducing silicon carbide wide-bandwidth semiconductor devices into hybrid DC circuit breakers, their high-frequency characteristics can effectively enhance the fault current’s cut-off speed. When deployed in a 200 kV hybrid DC circuit breaker, SiC devices enable an ultra-fast, 8 kHz current-injection stage. This speed reduces the fault current interruption time from 5 ms (the limit of Si-IGBT) to below 0.5 ms. Additionally, SiC devices offer 65% lower on-state losses and enable a 70% reduction in snubber volume. These advantages collectively reduce the overall breaker footprint by 1.7 times [73]. In turn, it provides an innovative technical path to solve the problems of high pass-state loss and complex system design of silicon-based devices, and it can help to promote the DC circuit breaker technology to realize significant development.

### 4.3. Power Electronics Transformers

Power electronic transformer (PET) refers to an intelligent power conversion device that integrates a power electronic converter and a high-frequency transformer. It can achieve voltage-level conversion, a two-way flow of energy and dynamic control of the power system by transforming the electric energy form and coupling with electromagnetic energy. Its working principle is that the power electronic transformer converts the input frequency alternating current into a direct current through a pulse width modulation (PWM) rectifier. And then, the high frequency inverter will be inverted to a high frequency alternating current. After utilizing a high-frequency transformer to achieve electrical isolation and voltage conversion, it is rectified to DC by a high-frequency rectifier. Finally, it can output suitable AC or DC power through an inverter or DC-DC converter, according to the load demand. The whole process relies on digital signal processing technology for real-time control of power electronic devices, so as to achieve the efficient conversion and management of electric energy.

The power electronic transformer reconstructs the traditional transformer mode through power electronics and a high frequency. It shows unique advantages in the field of the smart grid, microgrid, rail transportation, new energy grid connection, and so on. Compared with the traditional industrial frequency transformer, its core advantage is not only reflected in the significant reduction in volume and weight, but also with power quality optimization, power factor correction, fault current suppression, reactive power compensation, frequency conversion, phase conversion and intelligent monitoring and other multi-functions. Compared with a 50 Hz oil-immersed unit of the same 1 MVA 10 kV/400 V specification, PET prototypes exhibit a 6 kW·dm^−3^ power density and a 1.7 kW·kg^−1^ specific weight. This is corresponding to an 80–85% reduction in both volume and weight [74].

Current power electronic transformers have large switching losses. An elevated temperature under long-term operation may affect the life span. Quantitatively, PET switching losses increase by 60–140% when the junction temperature rises from 25 °C to 200 °C. Every 10 °C increase halves the semiconductor lifetime, so a 120 °C hotspot reduces insulation life from 20 years to 2.2 years [75,76]. Its average life expectancy is about 10 to 15 years, which is lower than the 20 to 30 years of conventional transformers.

However, a silicon carbide MOSFET provides new possibilities for technological innovation in power electronic transformers. Compared with a 6.5 kV Si-IGBT, a 3.3 kV SiC-MOSFET module reduces the turn-off energy Eoff from 25 mJ to 3 mJ (per 100 A), and raises the allowable junction temperature from 150 °C to 175 °C [77]. Silicon carbide power electronic transformers can support AC and DC multi-morphic power flexible access and bidirectional power flow. They are suitable for the dynamic regulation of the smart grid and enhance the ability for new energy consumption. Its three-phase integrated structure further compresses the volume under the same voltage capacity. Combined with high-frequency design, they achieve a double breakthrough in energy efficiency and power density. They can lay the technical foundation for the next generation of compact intelligent substation equipment. In the future, as the cost of wide-band semiconductors decreases topology optimization and the application of digital twin technology, the cost-effectiveness and reliability of PET is expected to be further improved. It will gradually replace some of the traditional transformer scenarios.

### 4.4. Flexible AC Transmission System

The flexible AC transmission system (FACTS) integrates the power electronic converter and control technology. It causes the continuous dynamic regulation and control of voltage amplitude, voltage phase, power quality and the power trend of AC transmission systems. It can significantly improve the capacity of AC transmission lines, reduce transmission losses and enhance system stability. Its working principle is based on the power electronic device combination of the FACTS controller. Through the precise control of single or multiple parameters of the transmission system, it upgrades the traditional passive and uncontrollable transmission network into a fully dimensional, controllable intelligent transmission network. In this way, we can enhance power system flexibility, security and reliability.

The static var compensator (SVC) and static synchronous compensator (STATCOM) are based on VSC and have the advantages of continuous adjustability, extremely fast response (microseconds) and compact size. They are mainly used for current control, dynamic reactive power compensation and system stability improvement. The working principle is to convert DC energy into AC energy through VSC. Real-time interaction with the grid creates bidirectional regulation and control of reactive power. Despite the simplicity of the two-level VSC-based topology, its high-voltage and large-capacity applications are limited by the voltage tolerance of silicon-based devices. Silicon carbide devices withstand an electric field that is 1.5 times higher (2.5 vs. 1.5 MV·cm^−1^) than silicon devices [78]. Meanwhile, a 6.5 kV Si-IGBT operates at 1 kHz and has a turn-off energy of about 1.2 J. By comparison, a 10 kV SiC module achieves a switching frequency of 5 kHz, with a turn-off energy of only about 30 mJ. These metrics translate directly to converter-level advantages. A 35 kV/±100 Mvar SiC-STATCOM, built with 10 kV MOSFETs, requires only three series devices per arm and achieves 99.2% efficiency at a 50% load. In contrast, its Si-IGBT counterpart needs six series devices and achieves only 98.1% efficiency [79]. Therefore, the expansion of the device’s voltage level needs to be realized by multiplexing transformer coupling or multilevel converter topology in engineering practice.

The above FACTS devices can further ensure the stable operation of the power system through the precise regulation of grid voltage and power flow. In view of the high-voltage and high-power application characteristics of the transmission network, early FACTSs mainly used thyristor, GTO and IGBT. However, IGCT technology is gradually maturing. With its 6.5 kV voltage withstand capability and 4000 A on/off current characteristics, it has gradually replaced GTO devices to build a new type of voltage source converter. It enables fast switching of high currents while increasing the voltage withstand level.

However, in the face of UHV transmission scenarios, it is still necessary to rely on multilevel topology or device series–parallel technology to further enhance the voltage withstand and current throughput capability. As a result, the reliability of the devices decreases, and the cost and losses increase significantly. Further engineering applications are deeply dependent on innovations in power electronics. The physical limits of silicon-based devices make them bottlenecks in higher voltage (±800 kV and above) or power (GW-class) FACTS controller applications. In this context, the development of new device structures and the adoption of SiC devices have become an inevitable path to break through the performance bottleneck.

As the voltage tolerance level of silicon carbide power devices continues to improve, their inherent high breakdown field strength and low switching-loss characteristics are becoming increasingly valuable in FACTS technology. Compared with traditional silicon-based IGBT power modules, SiC devices have significant advantages in high-frequency operating conditions. Their switching loss can be reduced by more than 50%. SiC MOSFETs reduce the total switching energy by 58%, versus Si IGBT in two-level topologies, and by 49% in MMC/ANPC. The actual energy saving varies with topology, gate resistance and stray inductance of the commutation loop, typically falling in the 45–68% range [80]. Together with efficient thermal management design, they can promote the development of devices in the direction of high efficiency, miniaturization and light weight. Therefore, they provide a new solution for the technological innovation of FACTS controllers.

## 5. Technical Bottlenecks and Future Research Directions

With the advancement of power electronics technology, the research and development of high-voltage silicon carbide power devices has gradually increased. Silicon carbide diodes at 19.5 kV, SiC GTOs at 4.5 kV, SiC MOSFETs at 10 kV and SiC IGBTs at 15 kV have been developed. At present, the widely used commercial SiC modules are mainly concentrated at 0.9~3.3 kV. Some of the voltage levels have been up to 6.5 kV. Devices at 10 kV and above are still in the R&D or early sampling phase. There is great potential for the application of silicon carbide devices in the field of high-voltage power transmission. There is huge potential for the application of silicon carbide devices in the field of high-voltage power transmission. However, high-capacity SiC devices with a high voltage and high current are still facing a series of key technical bottlenecks that need to be solved to realize their large-scale deployment.

(1) Insufficient high-voltage and high-current dynamic testing capabilities.

There is a lack of specialized test instruments for high-voltage (above 10 kV), high-current (thousands of amperes), and ultra-high switching speed (*dv*/*dt* > 50 kV/μs, *di*/*dt* > 10 kA/μs) SiC devices [81]. Existing commercial equipment has insufficient bandwidth, insufficient voltage/current ratings, and weak electromagnetic interference (EMI) immunity [82]. It is difficult to accurately and safely capture the real switching characteristics, loss distribution and failure modes of the devices under extreme operating conditions. This severely restricts device characterization, model validation and system design.

(2) Silicon carbide device packaging integration challenges.

Under a high power density, the low thermal conductivity of traditional packaging materials and the accumulation of thermal resistance leads to the junction temperature exceeding the limit. A single-sided heat dissipation path is difficult to match with the three-dimensional heat flow requirements. A thermal expansion coefficient mismatch between chip and package material and thermal stress during temperature cycling leads to cracking of the solder layer. A high temperature also accelerates the aging of the encapsulant. The thermal coupling is uneven in multi-chip integration. There is a contradiction in the insulation voltage of three-dimensional packages, which restricts the improvement of the power density [83].

(3) Gate oxygen reliability and interface stability issues.

The gate oxygen quality of SiC MOSFETs is a key bottleneck limiting their long-term reliability. Under high-temperature and high-electric-field operating conditions, excess carbon interstitials at the SiC/SiO_2_ interface aggregate into sp^2^ carbon clusters. These clusters introduce deep levels with a DOS peak of ~2 × 10^13^ eV^−1^·cm^−2^, locally enhancing the electric field [84]. Furthermore, the conductive clusters act as “hot-spot” channels, exceeding the electromigration threshold and reducing its activation energy from 0.8 eV to 0.55 eV [85]. This mechanism ultimately leads to a threshold voltage drift and transconductance degradation. Therefore, optimizing the gate oxide process and interface quality is essential for improving device reliability.

(4) Preparation of SiC substrate and the epitaxial layer.

The current substrate material has a high defect density, and the defects will be replicated in the epitaxial layer during the subsequent epitaxial growth process. The dominant defects are threading dislocations (TDs) and basal-plane dislocations (BPDs). Quantitative analysis reveals their respective densities: TDs measure 4.8 × 10^4^ cm^−2^, while BPDs measure 1.2 × 10^4^ cm^−2^ [86]. This results in a total defect density of approximately 6.0 × 10^4^ cm^−2^, which adversely affected the device’s performance by increasing the leakage current and lowering its blocking capability. Specifically, at 600 V, the leakage current rose from 0.8 μA to 5.2 μA, while the breakdown voltage fell from 2.1 kV to 1.4 kV [87]. It even induces the bipolar degradation phenomenon that is unique to silicon carbide. At the same time, the mechanism by which crystal defects degrade device performance remains unclear [88]. How different types of defects specifically affect the static parameters, dynamic switching characteristics and long-term reliability of the device still require systematic and in-depth research.

(5) Research and development of high-voltage, high-current silicon carbide devices.

Crystal defects within the silicon carbide substrate will significantly reduce the breakdown field strength. As a result, it is difficult to fully realize the theoretical withstand voltage, and the internal structure of the device process’ precision is easy to trigger the edge of the electric field concentration, resulting in early breakdown [89]. In order to balance the thick drift layer and low on-resistance required for high withstand voltage, the doping concentration needs to be increased [90]. However, this will exacerbate the problem of current unevenness at the polycrystalline interface. At the same time, electron mobility saturation at high fields limits further current density enhancement.

(6) High-voltage series connection and multi-chip parallel connection technology.

The faster switching speed of SiC devices makes them more sensitive to the effects of parasitic parameters. When multiple medium voltage SiC modules are connected in a series to achieve higher voltage levels, the transient voltage imbalance problem during the switching process will be more serious. There is an urgent need to develop active or passive dynamic voltage equalization strategies. The parallel connection of multiple chips can further create high-capacity applications. However, the inherent dispersion of SiC chip parameters and the extremely low thermal resistance path inside the module make it much more difficult to achieve static and dynamic current equalization [91]. Serious challenges are posed for current equalization and thermal coupling.

To address these bottlenecks and promote the widespread application of SiC devices in high-voltage power transmissions, future research should focus on these directions through multidisciplinary collaborative innovation. It is necessary to systematically solve the key technical problems, from materials and devices to packaging integration. It is also required for the advantages of SiC devices to lead the power transmission equipment toward the direction of high-frequency, high-temperature, high-efficiency and high power-density change.

## 6. Conclusions

This study systematically categorizes and summarizes the classification and characteristics of power electronic devices at different stages of energy development. It focuses on analyzing the application of SiC devices in high-voltage power transmission key equipment, such as flexible DC transmission systems, power electronic transformers, high-voltage circuit breakers and flexible AC transmission systems. Compared with silicon-based devices, the switching loss of high-voltage power transmission and transformation equipment, based on silicon carbide devices, can be reduced by 60–80%. The research also points out that the current high-voltage high-end power transmission and distribution equipment based on SiC devices is still facing key technical bottlenecks, such as cost, large-size wafer manufacturing, and the reliability of high-voltage module packaging. SiC, as a representative of the new generation of wide-bandwidth power electronic devices, is in a period of rapid development. Its excellent material properties will strongly promote power transmission and distribution equipment to a smaller-sized, higher-frequency, higher-temperature, higher-efficiency, higher-power-density direction of revolutionary evolution. This will profoundly change the architecture and operation mode of modern power systems. In order to fully release its potential, in the future, there needs to be continuous and in-depth research in the areas of device reliability improvement, scale-up cost control, high-temperature and high-power density package integration, intelligent drive protection strategy and system-level application verification.

## Figures and Tables

**Table 1:** Key parameters of semiconductor materials.

Material	Bandwidth E_g_ (eV)	Critical Breakdown Field E_F_ (MV/cm)	Electron Mobility μ (cm^2^/V·s)	Electron Saturation Drift Rate v (10^7^ cm/s)	Heat Conductivity λ (W/cm·K)	Dielectric Constant ε
Si	1.1	0.3	1350	1	1.5	11.8
3C-SiC	2.2	2.1	1000	2	4.5	9.6
4H-SiC	3.26	3.2	950	2	4.5	9.7
6H-SiC	3.0	3.0	500	2	4.5	9.7
GaN	3.39	3.3	900	2.5	1.3	9
Ga_2_O_3_	4.7	8	300	2.42	0.13	10
Diamond	5.45	5.6	1900	2.7	20	5.5

**Table 2:** Characteristics and application areas of SiC devices.

SiC Devices	Main Applications	Advantages
JBS Diode	Photovoltaics Electric vehicles Wind energy	No reverse recovery voltage
PiN Diode	DC transmission High voltage applications	High voltage and high current fields
MOSFET	Industrial power supplies Specialty applications	Gate drive weak regulation capability
JFET	Electric vehicles Wind power generation	High reliability
IGBT/GTO	High-speed rail traction DC power transmission	Ultra-high voltage and high current applications

## Data Availability

No new data were created or analyzed in this study.
